# Development of an *Aus*-Derived Nested Association Mapping (*Aus*-NAM) Population in Rice

**DOI:** 10.3390/plants10061255

**Published:** 2021-06-21

**Authors:** Justine K. Kitony, Hidehiko Sunohara, Mikako Tasaki, Jun-Ichi Mori, Akihisa Shimazu, Vincent P. Reyes, Hideshi Yasui, Yoshiyuki Yamagata, Atsushi Yoshimura, Masanori Yamasaki, Shunsaku Nishiuchi, Kazuyuki Doi

**Affiliations:** 1 Graduate School of Bioagricultural Sciences, Nagoya University, Chikusa-ku, Furo-cho, Nagoya 464-8601, Japan; kitony.kipruto.justine@g0.nagoya-u.jp (J.K.K.); taichung65@yahoo.co.jp (H.S.); tasakimikako21@gmail.com (M.T.); mjun.turuturu@gmail.com (J.-I.M.); pc0wc3874w2vj052x@yahoo.co.jp (A.S.); reyes.vincent.pamugas@f.mbox.nagoya-u.ac.jp (V.P.R.); s_nishi@agr.nagoya-u.ac.jp (S.N.); 2 Environmental Control Center Co., Ltd., Hachioji 193-0832, Japan; 3 Faculty of Agriculture, Kyushu University, 744 Motooka, Fukuoka 819-0395, Japan; hyasui@agr.kyushu-u.ac.jp (H.Y.); yoshiyuk@agr.kyushu-u.ac.jp (Y.Y.); ayoshi@agr.kyushu-u.ac.jp (A.Y.); 4 Food Resources Education and Research Center, Graduate School of Agricultural Science, Kobe University, Kasai 675-2103, Japan; yamasakim@tiger.kobe-u.ac.jp

**Keywords:** *aus*-NAM, GBS, QTL, *Ehd1*, *Hd1*, *Ghd7*, joint linkage mapping, GWAS

## Abstract

A genetic resource for studying genetic architecture of agronomic traits and environmental adaptation is essential for crop improvements. Here, we report the development of a rice nested association mapping population (*aus*-NAM) using 7 *aus* varieties as diversity donors and T65 as the common parent. *Aus*-NAM showed broad phenotypic variations. To test whether *aus*-NAM was useful for quantitative trait loci (QTL) mapping, known flowering genes (*Ehd1*, *Hd1*, and *Ghd7*) in rice were characterized using single-family QTL mapping, joint QTL mapping, and the methods based on genome-wide association study (GWAS). *Ehd1* was detected in all the seven families and all the methods. On the other hand, *Hd1* and *Ghd7* were detected in some families, and joint QTL mapping and GWAS-based methods resulted in weaker and uncertain peaks. Overall, the high allelic variations in *aus*-NAM provide a valuable genetic resource for the rice community.

## 1. Introduction

Improvement of rice (*Oryza sativa* L.) production has been achieved by development of new varieties and optimization of cultural practices. The majority of phenotypic variations of agricultural traits is determined by many loci with small effects (quantitative trait loci, QTL). Understanding the genetic basis of quantitative traits is essential for crop improvement. Application of DNA markers has become a common method in breeding programs. In addition, the recent advances in DNA marker technologies have enabled a systematic approach to identification of QTL and marker-assisted selection [1]. In rice, genome sequencing has enhanced identification of causal genes related to yield [2]. Most of these genes/QTL were identified using bi-parental populations, combined with the development of backcrossed progeny [3]. The backcross progeny enables precise estimation of allelic effects and fine-mapping of the target loci. However, limited allele richness is a disadvantage in these types of mapping populations.

On the other hand, development of next-generation sequencing (NGS) technology enabled direct detection of genetic loci associated with traits through genome-wide association studies (GWAS) [4]. In plants, GWAS can utilize a variety of germplasm collections such as landraces, breeding lines, and varieties that have accumulated recombination events both recent and historic. This attribute gives GWAS a higher gene mapping resolution compared with bi-parental populations [5,6,7]. However, unaccounted population structures/stratifications make GWAS prone to false associations. Additionally, the absence of pedigree information in diversity panels prohibits classical pedigree-based haplotype mapping, consequently reducing statistical power [8,9,10].

To combine the advantages of bi-parental populations and diversity panels, multi-cross mating designs consisting of diverse donors have been proposed. Examples of multi-cross designs include nested association mapping (NAM) [11], multi-parent advanced generation inter-crosses (MAGIC) [12], and random-open-parent association mapping (ROAM) [13]. Multi-parent populations have advantages, such as (1) allele richness coming from the diversity donors, (2) known population structures, and (3) flexibility to be used as a breeding platform.

The first NAM population was reported in maize [14]. The population was successfully used to profile the genetic architecture of agronomic traits, such as flowering time, leaf architecture, stalk strength, and plant height [15,16,17]. Following the successes in maize, NAM populations were developed in other crops, such as rice [18], wheat [19], barley [20], soybean [21], sorghum [22], and rapeseed [23].

Asian rice (*O. sativa*) is classified into five major varietal groups, namely, *temperate japonica*, *tropical japonica*, *indica*, *aus*, and *aromatic* [24]. *Aus* rice varieties are considered to have evolved from annual *Oryza nivara* found in Bangladesh, Northeast India, Nepal, and northern Myanmar. Most *aus* varieties exhibit photoperiod insensitivity, a source of local environmental adaptation. Moreover, *aus* is known to possess drought tolerance and rice blast resistance [7,25,26,27].

In this study, we describe the development of a rice NAM population using *aus* varieties as diversity donors (*aus*-NAM) and test its QTL mapping powers using known flowering-time genes. The set of recombinant inbred lines (RILs) utilized was generated using single-seed descent. Genotypes of each line were determined using NGS. For GWAS, parental DNA variants were projected onto the progeny genotypes, and mapping of days to heading (DTH) QTL/genes was performed.

## 2. Results

### 2.1. Development of Aus-NAM Population

Out of the seven families used in this study, five (WNAM02, WNAM29, WNAM31, WNAM35, and WNAM39) were newly developed. The numbers of plants in F_2_ and F_5_ are listed in Table 1. Because of hybrid sterility and late heading, a substantial number of the plants in F_2_, F_3_, and F_4_ could not be harvested. The residual rate for families at F_5_ ranged from 46.8% to 74.4% (Table 1). In total, 895 RILs ranging from 107 to 163 per family were obtained (Table 1).

### 2.2. Phenotypic Characteristics

The mean values of DTH in each family varied from 88 days to 105 days with potential transgressive segregation in some families, and some of the lines showed extreme values compared to the parents (Figure S1). Analysis of variance showed a statistically significant difference among the RIL families with an F value of 73.52 and a *p*-value < 2 × 10^−16^.

### 2.3. Linkage Map and Projection of Parental Variants

The number of clean SNPs obtained from GBS in each family ranged from 2868 to 4285 (Table S1). Upon filtering lines with excess heterozygosity (>0.125), 887 RILs were retained for subsequent genetic analyses (Table S1). For QTL mapping, 1786 non-redundant SNP markers were used. They covered all the 12 rice chromosomes with an average distance between markers ranging from 0.41 cM to 0.86 cM (Table S2).

For GWAS, parental variants obtained from whole-genome resequencing (4,643,123 SNPs) were firstly filtered then projected onto each of the individual family skeleton linkage maps. A total of 41,561 SNPs were included in the association analysis.

### 2.4. Population Structure

**Figure 1:**
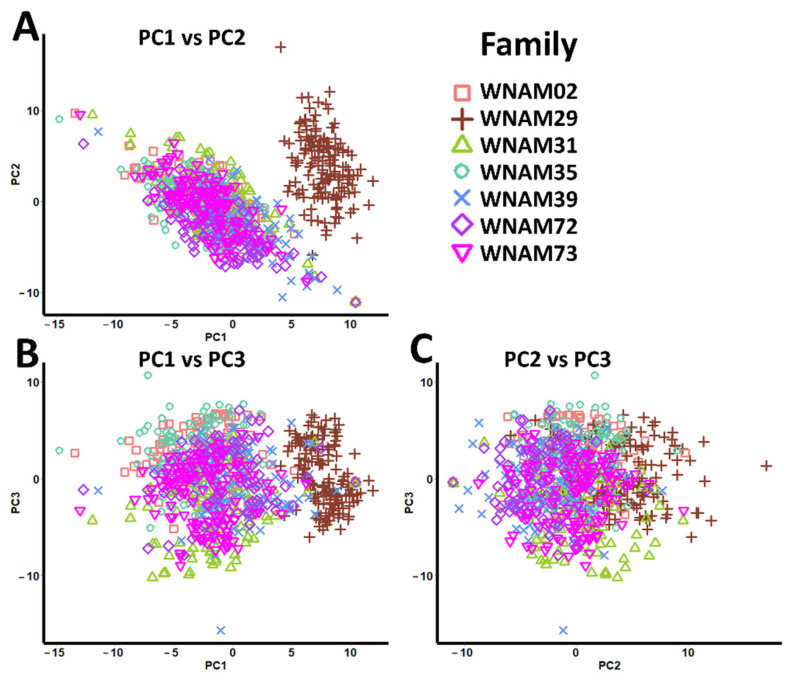
Probabilistic principal component analysis (PPCA) showing population structure in the *aus* nested association mapping (*aus*-NAM) population. (**A**) PC1 vs. PC2, (**B**) PC1 vs. PC3, and (**C**) PC2 vs. PC3. Different shapes and colors represent different *aus*-NAM families.

Estimation of population structure using probabilistic principal component analysis (PPCA) showed fairly controlled stratification. The R^2^ values were 0.067, 0.044, 0.041, 0.038, 0.037, and 0.034 for PC1, PC2, PC3, PC4, PC5, and PC6, respectively. (Figure 1, Figure S2, and Table S3). WNAM29 formed an isolated group from other families (Figure 1).

### 2.5. QTL Detected by Single-Family Analysis, Joint Analysis, and GWAS-Based Methods

**Figure 2:**
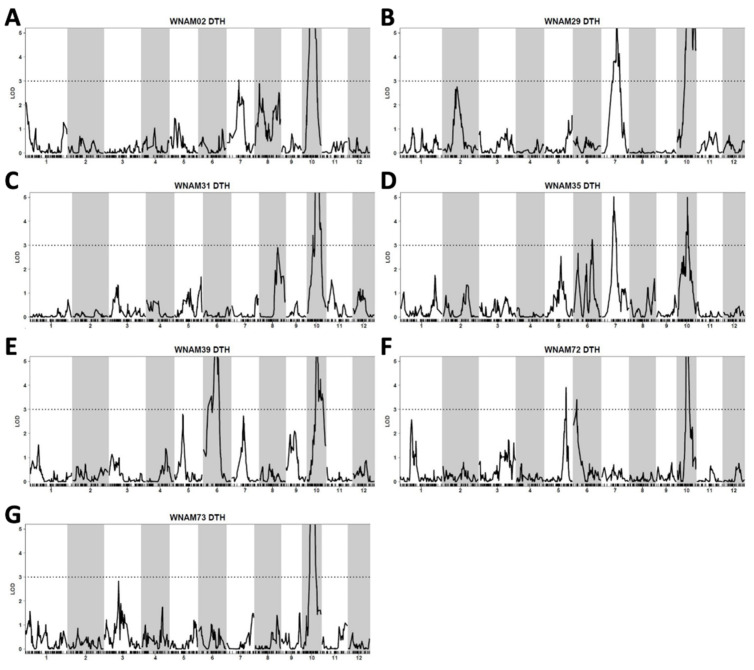
Interval mapping showing the locations of days to heading QTL in *aus* nested association mapping (*aus*-NAM) families using R/QTL scanone function. (**A**) WNAM02, (**B**) WNAM29, (**C**) WNAM31, (**D**) WNAM35, (**E**) WNAM39, (**F**) WNAM72, and (**G**) WNAM73. The black lines correspond to LOD score profile (Y-axis) as a function of distance in cM across each chromosome (X-axis). Horizontal dotted lines in all panels indicate the LOD threshold value of 3.

Single-family QTL analysis detected a total of 14 significant additive QTL on chromosomes 5, 6, 7, and 10 (Figure 2 and Table S4). The QTL individually explained 12% to 36% of the trait variances. Among the QTL detected, eight contained alleles increasing days to heading from *aus* and six from T65, respectively. The QTL on chromosome 10 was detected across all families, with WNAM73 possessing the highest LOD score (11.81) (Table S4).

**Figure 3:**
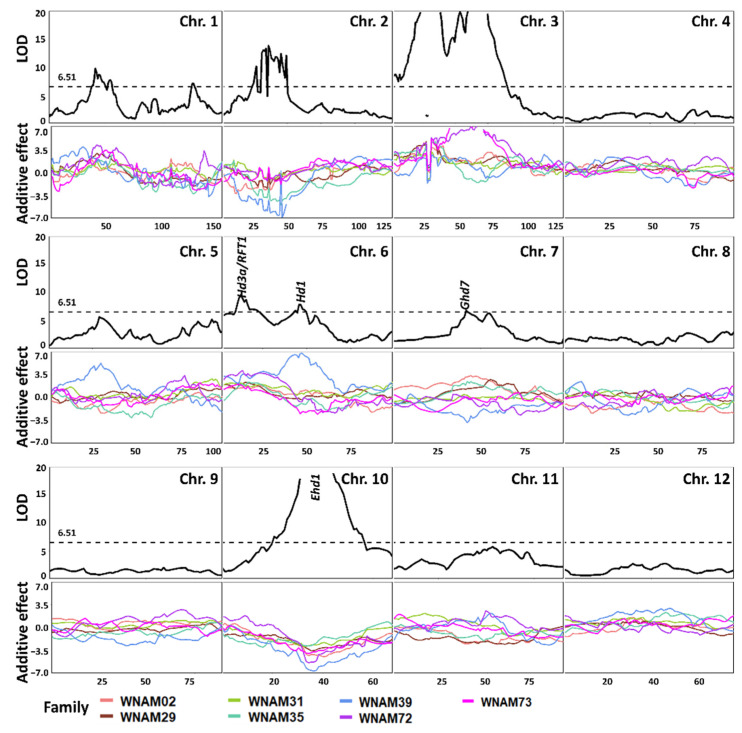
LOD profile of QTL (top of each panel) and its additive effects (bottom of each panel) detected by joint inclusive composite interval mapping (JICIM). Scanning step was 1 cM, and the dotted horizontal line (6.51) represents thresholds obtained from 1000 permutation tests with type I errors of 0.05. Different line colors in additive effect panels represent different families. The positions of known loci (*RFT*, *Hd3a*, *Hd1*, *Ehd1*) are shown in the panels of chromosomes 6, 7, and 10.

Joint linkage mapping identified a total of 19 QTL (Figure 3 and Table S5). Some of the joint QTL overlapped with single-family QTL, such as QTL on chromosomes 6, 7, and 10. The peak on chromosome 10 was detected as a single peak. The peaks on chromosomes 6 and 7 appeared to combine peak locations of the seven populations, resulting in two separate peaks on each chromosome. In addition, 14 putative QTL spanning a relatively wide region were detected on chromosomes 1, 2, and 3 (Figure 3). On the other hand, a significant QTL on chromosome 5 in WNAM72 (Figure 2F) was not detected in the joint QTL analysis.

**Figure 4:**
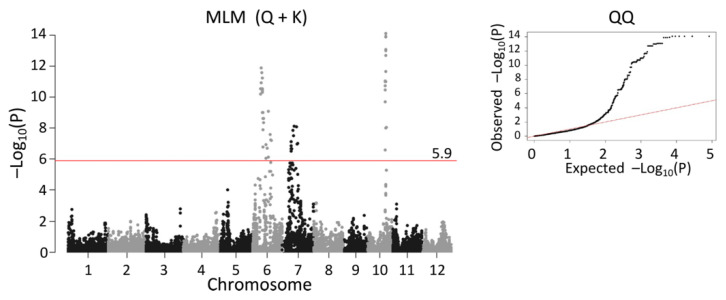
Manhattan plot of days to heading using mixed linear model with principal components (Q) and genomic kinship (K) as covariates (MLM (Q + K)). The red horizontal line marks the threshold for genome-wide significance (5.9) on a −log_10_ scale. A quantile–quantile (QQ) plot is shown in the right panel, where the observed *p*-values (Y-axis) against the expected *p*-values (X-axis) under the null hypothesis of no association are plotted on a −log_10_ scale. Each black dot indicates an SNP.

Association mapping by a naive general linear model revealed significant QTL signals in all chromosomes (Figure S3), while mixed linear model analysis identified significant SNPs on chromosomes: 6, 7, and 10 (Figure 4), and a total of 188 SNPs met the negative logarithm P value (−Log_10_P) of 5.9 Bonferroni threshold at alpha 0.05.

### 2.6. Evaluation of Mapping Accuracy

The QTL commonly detected in the three methods on chromosomes 6, 7, and 10 included the regions of *RFT1* [29], *Hd3a* [30], *Hd1* [31], *Ghd7* [32], and *Ehd1* [33]. Assuming that *Hd1*, *Ghd7*, and *Ehd1* were the genes underlying the detected QTL, these loci were used to evaluate the accuracy of gene mapping using *aus*-NAM.

**Figure 5:**
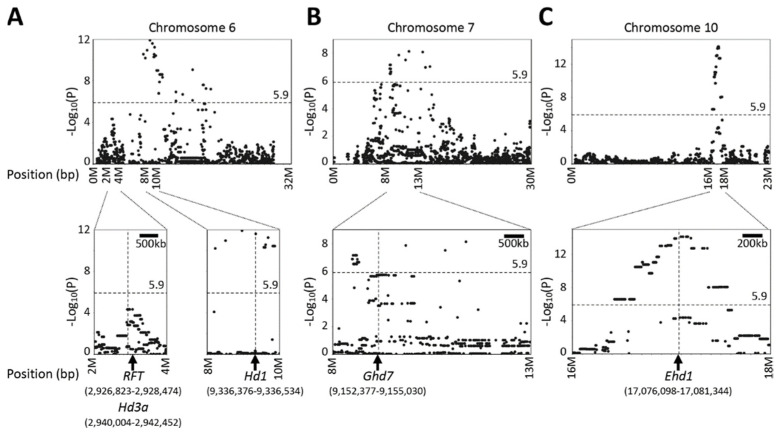
Manhattan scatter plots showing local association of days to heading in the *aus* nested association mapping (*aus*-NAM) population: (**A**) chromosome 6, (**B**) chromosome 7, and (**C**) chromosome 10. The panels at the bottom are magnification around *RFT/Hd3a*, *Hd1*, *Ghd7*, and *Ehd1* loci.

A major QTL on chromosome 10 was identified in every individual family (Figure 2 and Table S4). This QTL corresponded to *Ehd1* (Os10g0463400) [33], which is located between 17,076,098 bp and 17,081,344 bp on chromosome 10. The peak was detected at the marker position from 16,626,134 bp to 17,367,103 bp in single-family QTL mapping (Figure 2), while joint QTL analysis identified marker position 16,772,764 bp as the peak (Figure 3). MLM (Q + K) detected a peak spanning from 17,095,439 bp to 17,164,368 bp (Figure 5C). Thus, all of the statistical methods successfully detected *Ehd1*.

A significant QTL was detected in WNAM39 (Badari Dhan), close to *Hd1* on chromosome 6 (9,335,377 bp to 9,337,570 bp) (Figure 2E). This peak was flanked by the markers S06_8837126 and S06_9318022 in joint linkage analysis with an LOD score of 7.84 (Figure 3). In MLM (Q + K), marker S06_9338330 was the most significant (−Log_10_P value of 11.6) (Figure 5A). To better understand the individual family contributions to the joint peak on chromosome 6 (Figure 3), additive effects at this locus were further analyzed. The Badari Dhan allele had the highest additive effect value (6.72 days) (Table S5). Multiple alignment of the amino acid sequences deduced using the genomic sequences confirmed loss-of-function in *aus* varieties except for Badari Dhan (Figure S4). Another signal in the vicinity of *RFT1* and/or *Hd3a* on chromosome 6 was also detected by GWAS (Figure 4 and Figure 5), although the signal did not reach the significant threshold.

*Ghd7* is located between 9,184,534 bp and 9,187,187 bp on chromosome 7 [32,34]. Single-family QTL analysis showed that WNAM02 and WNAM35 families had a significant QTL in the vicinity of *Ghd7* (Figure 2A,D). Therefore, it was hypothesized that Kasalath (WNAM02) and ARC5955 (WNAM35) possess functional (late) alleles for *Ghd7*. The peak corresponding to *Ghd7* in joint QTL was from 8.93Mbp to 9.35Mbp on chromosome 7, which contained the *Ghd7* locus (Figure 3). MLM (Q + K) detected a cluster of markers around the *Ghd7* locus with −Log_10_P values greater than 5, surrounded by further markers that showed higher −Log_10_P values than the threshold. Thus, the position of *Ghd7* was not clear from this analysis. In WNAM39, a QTL near *Ghd7* was detected where the *aus* allele had a negative additive effect that was opposite from other families, and this peak was also detected in the joint QTL analysis.

## 3. Discussion

### 3.1. Development of aus-NAM Population

The NAM population brings the advantage of being able to apply both joint linkage analysis and GWAS in dissecting the genetic basis of complex traits [11,14,16]. In this study, we developed and characterized the first rice NAM population using the *aus* varietal group as diversity donors. Because of hybrid sterility and late heading, a substantial number of lines were lost in the process of single seed descent. However, the population size allowed detection of both known and novel QTL (Figure 2).

While examining the population structure of *aus*-NAM, PPCA showed only a weak stratification, with half-sib RILs dispersed around the T65 (common female parent) and *aus* (diversity donor parent) (Figure 1 and Figure S2). A similar population structure was reported in oilseed rape NAM [23] and sorghum NAM [22]. This confirmed that *aus*-NAM retained the genetic diversity of donors, and the population structure was suitable for analyzing the genetic architecture of complex traits. Further expansion of the seven *aus*-NAM RIL populations will fill the gaps and enhance the statistical power of *aus*-NAM.

### 3.2. Genotyping of aus-NAM Population

A GBS method [35,36] was used to obtain marker genotypes of *aus*-NAM. The potential number of markers obtained by the restriction enzyme used in this study (*Kpn*I-*Msp*I) was estimated at approximately 5000 loci. This number is sufficient to model phenotypes based on best linear unbiased prediction [37], hence, enough for gene mapping. Therefore, the choice of the enzyme was appropriate for *aus*-NAM. The advancement of next-generation sequencing technology will allow the use of more “frequent” cutters instead of *Kpn*I or even whole-genome shotgun for all of the lines.

### 3.3. Accuracy of QTL Mapping Using aus-NAM Population

To date, over 40 flowering QTL have been identified in rice [34]. In this study, *Ehd1* [33] was detected as the most common major QTL. The mapped positions of the QTL on chromosome 10 corresponded to the actual position of *Ehd1* (Figure 2, Figure 3, Figure 4, and Figure 5C). Another heading time locus, *Hd1* [31], was detected only in WNAM39, and analysis of the deduced amino acid sequence confirmed that the founder of WNAM39 (Badari Dhan) was the only variety possessing the functional allele of *Hd1* (Figure S4). The effect of a functional allele of *Hd1* in the environment of this study (long day) was to delay heading, and it matched the observed result. However, the *Hd1* peak in Figure 5A was not surrounded by markers with smaller –Log_10_(P) values such as *Ehd1*. This was probably because the sequence differences between Badari Dhan signals of linked markers were diluted by other families. Without prior information, *Hd1* would not be mapped to a precise position using MLM (Q + K).

Unlike *Ehd1* and *Hd1*, it was not possible to discriminate alleles at *Hd3a*, *RFT1,* and *Ghd7* despite the previous reports [29,30,32]. However, it should be noted that joint QTL precisely mapped the peaks of *Ehd1*, *Hd1*, *Ghd7*, and a combined peak of *RFT1*/*Hd3a* (Figure 3). The joint QTL mapping approach has been reported to amplify small effects signals found on individual family RILs [18]. The results in the present study indicated that joint QTL mapping is advantageous in the precision of QTL positioning compared with MLM (Q + K).

A QTL tightly linked to *Ghd7* was detected in WNAM02 and WNAM35. Another QTL near, but not tightly linked to *Ghd7* (11.0Mb on chromosome 7, 2.08Mb apart from *Ghd7* (8.93Mb)), was detected in WNAM39, where the functional allele of *Hd1* segregated. This QTL showed an opposite additive effect as that expressed in WNAM02 and WNAM35. *Ghd7* was reported to have the ability to switch its additive effects by an epistasis with *Hd1* [38]. The underlying gene in the QTL detected in WNAM39 remains unclear. A well-saturated linkage map will facilitate the characterization of genes underpinning this QTL.

### 3.4. Prospects

In the modeling used for genomic selection in practical breeding programs, population size is more important than marker density [37]. Further expansion of the population being conducted by the authors will enable accurate association analysis by eliminating the limitations of population size. The new population, with more founders, will have a higher representation of rare alleles, which is important for crop improvements [39,40,41]. In addition, the immortal nature of NAM populations allows for evaluations in various environments. Thus, they could be used to reveal heading time gene networks in detail. In conclusion, our results elucidated that *aus*-NAM will be a valuable genetic resource for QTL mapping and may be used for genomic selection.

## 4. Materials and Methods

### 4.1. Plant Materials

An *aus*-NAM was built using a temperate japonica variety, Taichung 65 (T65), as the common female parent. The five *aus* cultivars Kasalath, Kalo Dhan, Shoni, ARC5955, and Badari Dhan (kindly supplied by the National Agricultural Research Organization (NARO) Genebank, Tsukuba, Japan) were used as diversity donor parents (founders) [28]. The five *aus* varieties were crossed to T65, and RILs were derived from the F_2_ generation using single-seed descent (SSD) to obtain F_5_ in 2015. The RILs (F_13_) from T65 x DV85 and T65 × ARC10313 were generated at Kyushu University, Fukuoka, Japan, using SSD, and were kindly provided through National Bioresource Project. The 7 families of RILs were designated as WNAM02 (Kasalath), WNAM29 (Kalo Dhan), WNAM31 (Shoni), WNAM35 (ARC5955), WNAM39 (Badari Dhan), WNAM72 (DV85), and WNAM73 (ARC10313).

### 4.2. Trait Evaluation and Statistical Analysis

Field trials were conducted in 2015 at Togo Field, Nagoya University, Aichi, Japan (35°06′36.5″ N, 137°05′06.3″ E). Twelve to fifteen seeds per line of F_5_ were sown on 27 May, and four plants per line were transplanted on 2 July, in a randomized design without replication in a single plot. The spacing was 20 cm between the hills and 30 cm between rows. Standard agronomic management was followed during the trials, except no fertilizer was applied.

Days to heading (DTH) was calculated as the difference between the date of emergence of inflorescence and sowing. The average standard deviation within a line was approximately 2.64 days; thus, most of the lines can be regarded as genetically fixed. The second plant in each line was used for both of phenotyping and genotyping. Phenotype value distributions across subpopulations were examined. To find which trait means were significantly different among *aus*-NAM families, a one-way analysis of variance (ANOVA) followed by Tukey HSD with a 95% confidence level was performed. All statistical analyses and visualizations were performed using R version 4.0.3 [42].

### 4.3. Genotyping By Sequencing (GBS)

For genotyping, approximately 5 cm of leaf tissues from each line was sampled into paper envelopes. The samples were dried in an oven at 53 °C overnight and then stored at 6 °C. Total DNA of RILs and founders were extracted using a modified Dellaporta method [43]. DNA qualities were checked by electrophoresis on a 0.6% agarose gel in 1 × Tris/Borate/EDTA (TBE; 40 mmol/L Tris, 20 mmol/L acetic acid, and 0.5 mmol/L EDTA-2Na). The Quantiflour dsDNA system (Promega, Madison, WI, USA) was used for the quantification of the extracted total double-stranded DNA. GBS libraries were prepared using reported protocols [35,36]. Briefly, 200 ng (20 ng × 10 µL) of individual samples of DNA was double-digested with *KpnI* and *MspI* enzymes (New England Biolabs Inc., Ipswich, MA, USA), ligated to barcode adaptors, pooled (multiplexed), and purified using a QIAquick PCR Purification Kit (Qiagen Sciences, Germantown, MD, USA). Flowcell primers were added to the pooled samples and amplified. The library was sequenced using Illumina MiSeq (Illumina, San Diego, CA, USA).

Raw sequences were processed using the TASSEL-GBS pipeline [44] with default parameters, except (1) minimum allele frequency higher than 0.02, (2) minimum locus coverage set to 0.3, and (3) heterozygous sites and taxa that exceeded 0.125 were filtered out. Os-Nipponbare-IRGSP-1.0 [45] was used as the reference for SNP identification. SNPs were further filtered based on parental polymorphism. Only loci that were polymorphic between parents but monomorphic in each parent were included. Additionally, missing data were imputed using the FSFHap algorithm [46].

### 4.4. Whole-Genome Resequencing of T65 and aus Founders

DNA of the founders was extracted using the cetyl trimethylammonium bromide (CTAB) method, then fragmented using Covaris Model S2 (Covaris, Woburn, MA, USA) and used to construct a sequencing library using a TruSeq DNA LT kit (Illumina, San Diego, CA, USA). Sequencing was conducted using Illumina Miseq with a Miseq Reagent Kit v3 (600 cycles). Variant calling was conducted following the standard protocol of the Genome Annotation ToolKit (GATK) [47] using Os-Nipponbare-IRGSP-1.0 [45] as the reference.

### 4.5. Projection of Parental Variants and Population Structure Estimation

SNPs from parental read sequences were projected onto each RIL. Projections were performed in two steps: (1) employ GBS markers as skeletons and (2) check adjacent skeleton markers. If homozygous and possessing the common allele type as one of the parents, project the parental SNPs onto the intervals; otherwise, set the intervals as missing. Population stratifications were estimated using a probabilistic PCA (PPCA) algorithm in the Bioconductor package PCA methods [48] implemented in R.

### 4.6. Simple QTL Mapping

Genotype files in hapmap format were converted to parent-based format, where A represented T65 and B represented *aus* genotype; heterozygotes and missing were represented by H and “-”, respectively. Kosambi mapping function in R/qtl package [49] was used to obtain genetic distances in cM. QTL mapping was performed based on interval mapping using the “hk” method implemented in R/qtl. The additive effects of a marker were calculated as ((average of *aus*) − (average of T65))/2. Positive and negative additive effects values implied that *aus* and T65 alleles increased trait values respectively. A logarithm of odds (LOD) value of 3 was used as the threshold, while 3.04 was obtained as the empirical threshold (type I error of 0.05) based on 1000 permutation tests [50].

### 4.7. Joint QTL Mapping

For joint linkage mapping, genotype information in a common genetic map and DTH were subjected to joint inclusive composite interval mapping (JICIM) [51]. Missing phenotypes were replaced by the mean of the trait, 1 cM step was selected, and LOD threshold was obtained from 1000 permutation tests with a type I error of 0.05. The genotype file was converted into a numeral format where the T65 genotype was represented by 0, and *aus* genotype was represented by 2; heterozygous genotypes and missing genotypes were represented by 1 and −1, respectively. Positive and negative additive effect values mean that *aus* and T65 alleles increased trait values respectively.

### 4.8. Genome-Wide Association Analysis

TASSEL (trait analysis by association, evolution, and linkage) software [52] was used for QTL mapping using a GWAS-based method. General linear model (GLM) and mixed linear model (MLM) with principal components (Q) to account for population structure and genomic kinship (K) as covariates (MLM (Q + K)) were used. In MLM (Q + K), pedigree information (family) was used by TASSEL software as covariates. The threshold for declaring significance was determined using Bonferroni with the equation: *p* ≤ 1/N (α = 0.05), where N is the number of markers [53].

## Figures and Tables

**Table 1:** List of recombinant inbred lines in the *aus* nested association mapping population.

Family Name	Founder’s Name	WRC No. ^1^	F_2_	F_5_	Residual Rate
WNAM02	Kasalath	WRC02	233	109	46.78%
WNAM29	Kalo Dhan	WRC29	219	163	74.43%
WNAM31	Shoni	WRC31	174	121	69.54%
WNAM35	ARC5955	WRC35	229	137	59.83%
WNAM39	Badari Dhan	WRC39	213	126	59.15%
WNAM72	DV85	-	-	107	-
WNAM73	ARC10313	-	-	132	-

^1^ World Rice Core Collection number [28].

## Data Availability

*Aus*-NAM germplasm and genotypes are available by request via kdoi@agr.nagoya-u.ac.jp.

## Supplementary Materials

The following are available online at https://www.mdpi.com/article/10.3390/plants10061255/s1, Figure S1: Violin plots showing the frequency distributions for days to heading (DTH) in *aus* nested association mapping population. Yellow and blue dots represent the population founders, *aus* and T65 respectively. Black dots show the recombinant inbred lines average in each family. Groups with no significant difference by Tukey HSD with a 95% confidence level are represented by the same letters shown above the plots; Figure S2: Probabilistic principal component analysis (PPCA) showing population structure in *aus*-NAM. (A) PC2 vs. PC3 (B) PC3 vs. PC4 (C) PC5 vs. PC6. Different shapes and colors represent different *aus*-NAM families; Figure S3: Manhattan plot of days to heading using general linear model (GLM). The red horizontal line marks the threshold for genome wide significance (5.9) on a −log10 scale. A quantile-quantile (QQ) plot is shown in the right panel, where the observed P-values (Y-axis) against the expected *p*-values (X-axis) under the null hypothesis of no association are plotted on a −log10 scale. Each black dot indicates an SNP; Figure S4: Alignment of amino-acid sequences of *Hd1* in functional alleles of Nipponbare and Ginbouzu, and Badari Dhan (WRC39). The amino acid sequences were deduced from genomic sequences. Regions of the 2 ZF-B box and CCT motif are indicated. The sequence of Badari Dhan contained 6 non-synonymous mutations, but it was considered that the allele retains function; Table S1: Number of SNP markers and lines in *aus* nested association mapping (*aus*-NAM) population; Table S2: Joint linkage map statistics in *aus* nested association mapping population; Table S3: *R2* values obtained by probabilistic principal component analysis (PPCA); Table S4: Days to heading QTL detected using single-family QTL analysis; Table S5: Days to heading QTL and additive effects detected using joint linkage analysis.
