# Supplementary material for: Development of an Aus-Derived Nested Association Mapping (Aus-NAM) Population in Rice
Source: Plants (Basel). 2021 Jun 21;10(6):1255. doi: 10.3390/plants10061255 (PMC8234321; doi:10.3390/plants10061255)
Supplement: Supplementary file 1 [file plants-10-01255-s001.zip › plants-Supplementary_Figures-1242180.pdf]

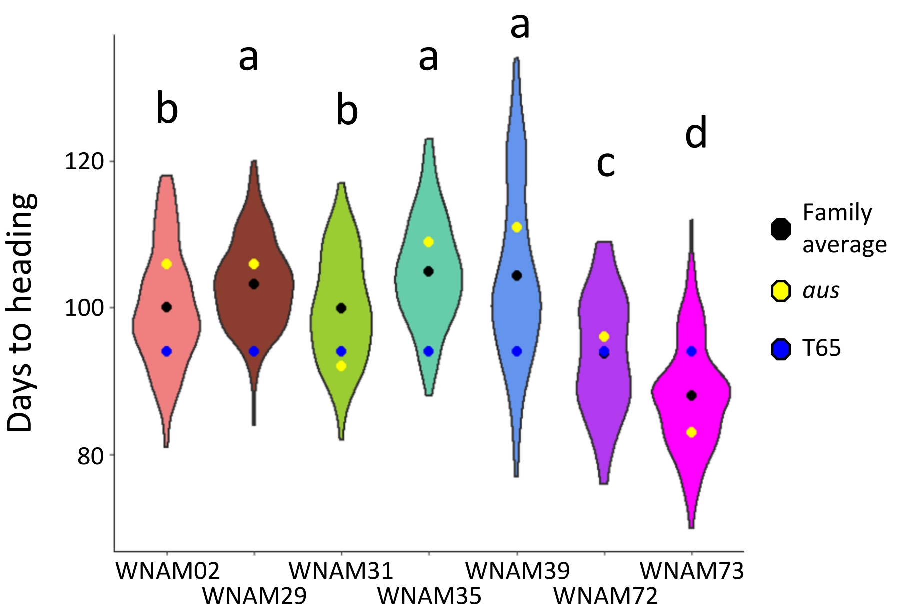

Figure S1. Violin plots showing the frequency distributions for days to heading (DTH) in *aus* nested association mapping population. Yellow and blue dots represent the population founders, *aus* and T65 respectively. Black dots show the recombinant inbred lines average in each family. Groups with no significant difference by Tukey HSD with a 95% confidence level are represented by the same letters shown above the plots.

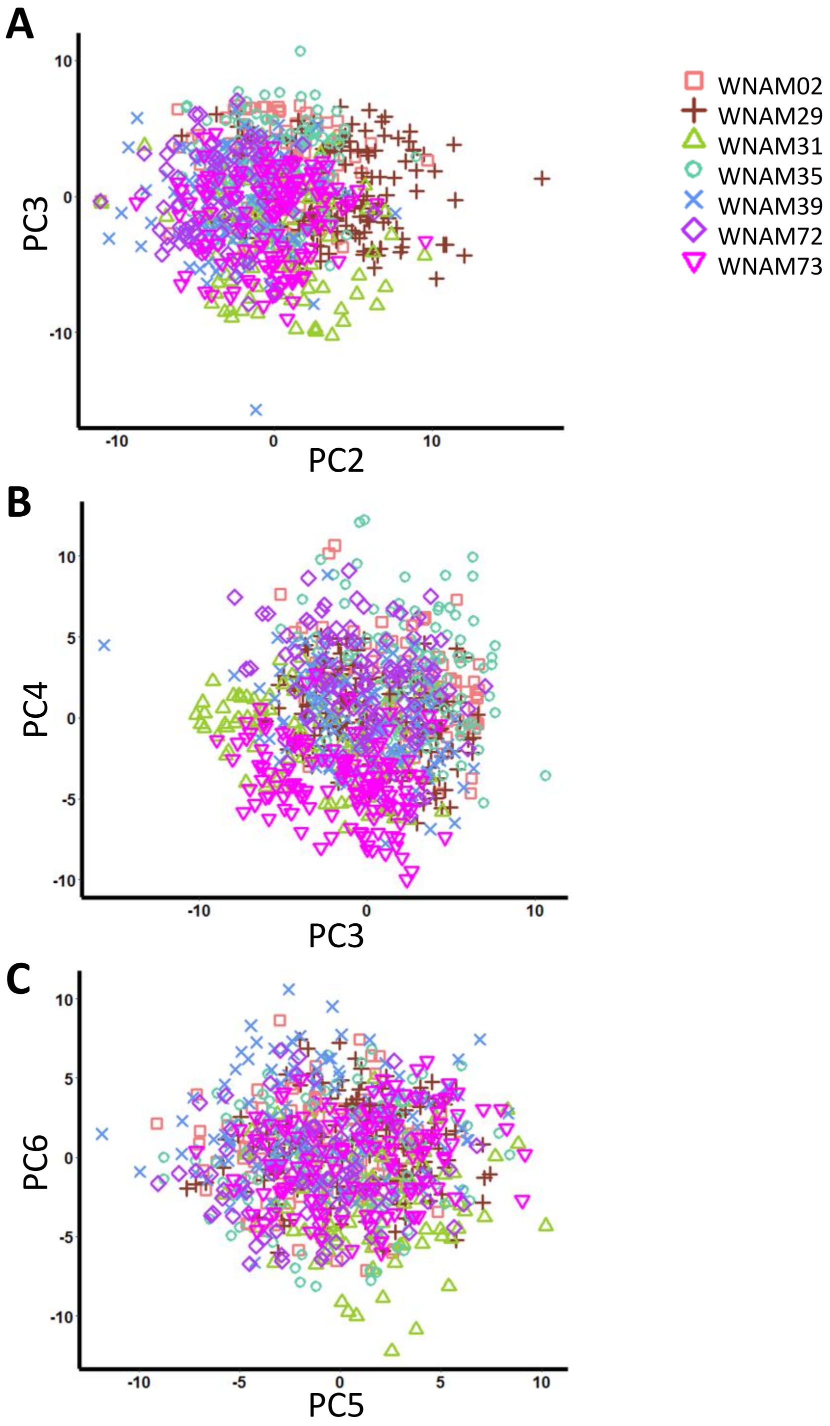

Figure S2. Probabilistic principal component analysis (PPCA) showing population structure in *aus* nested association mapping (*aus*-NAM) population. (A) PC2 vs PC3 (B) PC3 vs PC4 (C) PC5 vs PC6. Different shapes and colors represent different *aus*-NAM families.

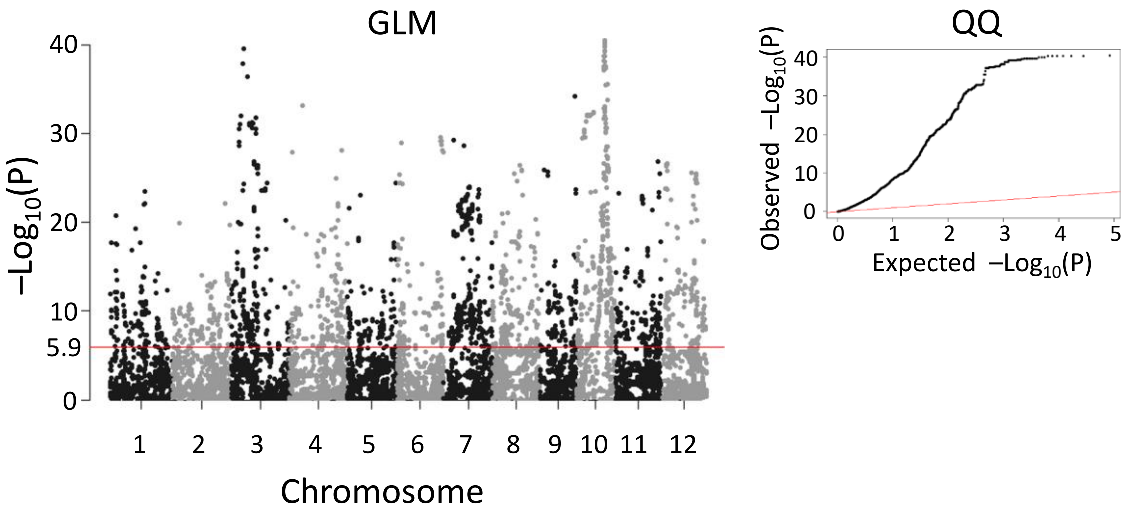

Figure S3. Manhattan plot of days to heading using general linear model (GLM). The red horizontal line marks the threshold for genome wide significance (5.9) on a -log10 scale. A quantile-quantile (QQ) plot is shown in the right panel, where the observed P-values (Y-axis) against the expected P-values (X-axis) under the null hypothesis of no association are plotted on a -log10 scale. Each black dot indicate a SNP.

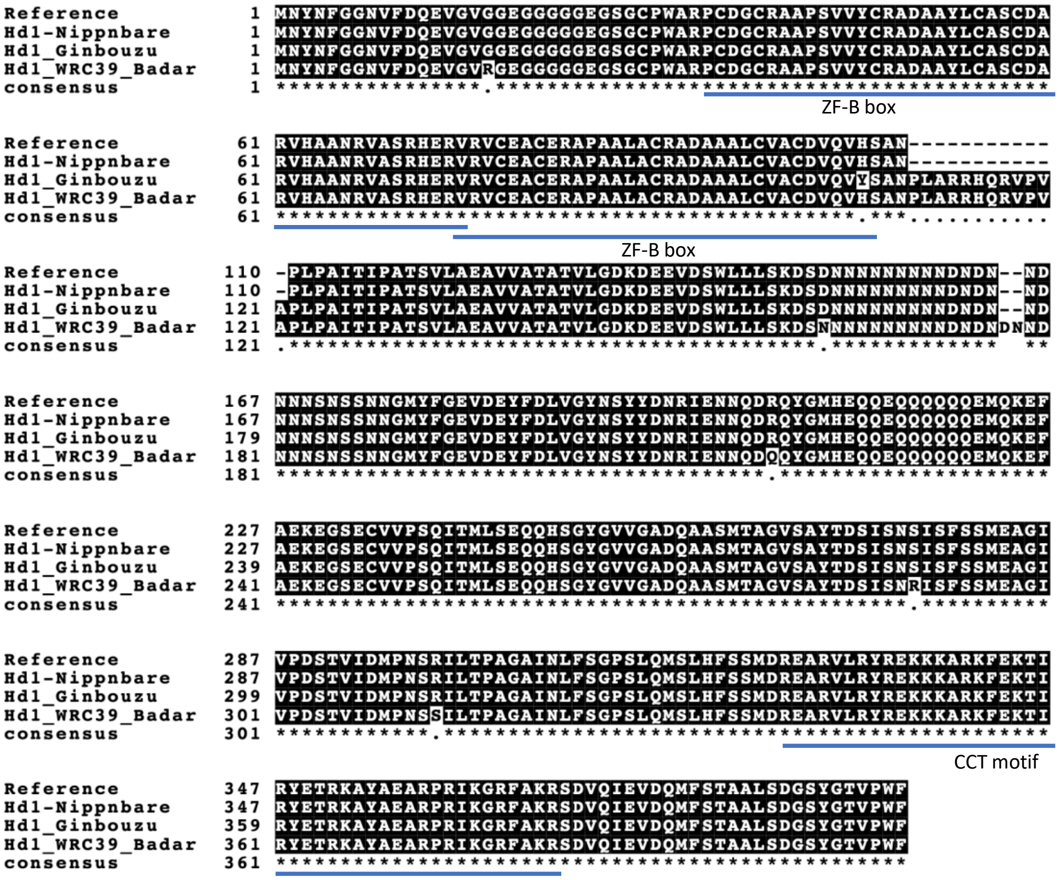

Figure S4. Alignment of amino-acid sequences of *Hd1* in functional alleles of Nipponbare and Ginbouzu, and Badari Dhan (WRC39). The amino acid sequences were deduced from genomic sequences. Regions of the 2 ZF-B box and CCT motif are indicated. The sequence of Badari Dhan contained 6 non-synonymous mutations, but it was considered that the allele retains function.
